# Supplementary figures and images for: Exploiting the Adaptation Dynamics to Predict the Distribution of Beneficial Fitness Effects
Source: PLoS One. 2016 Mar 18;11(3):e0151795. doi: 10.1371/journal.pone.0151795 (PMC4798746; doi:10.1371/journal.pone.0151795)

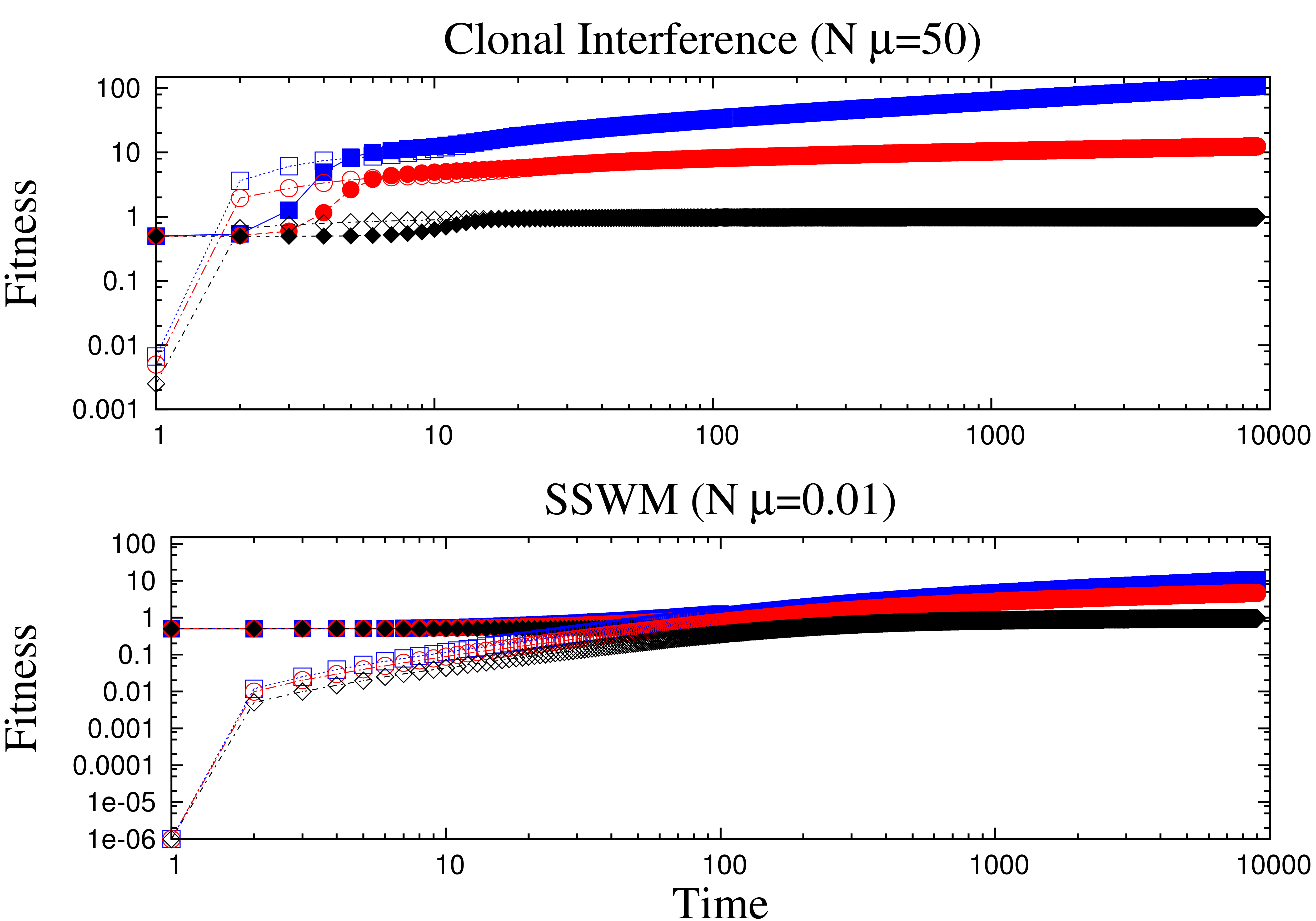

Supplement: S1 Fig — The lines give the theoretical values while the open symbols are the simulation output for Nμ = 0.02 and the closed symbols are those for Nμ = 5. (TIF) [file pone.0151795.s001.tif]

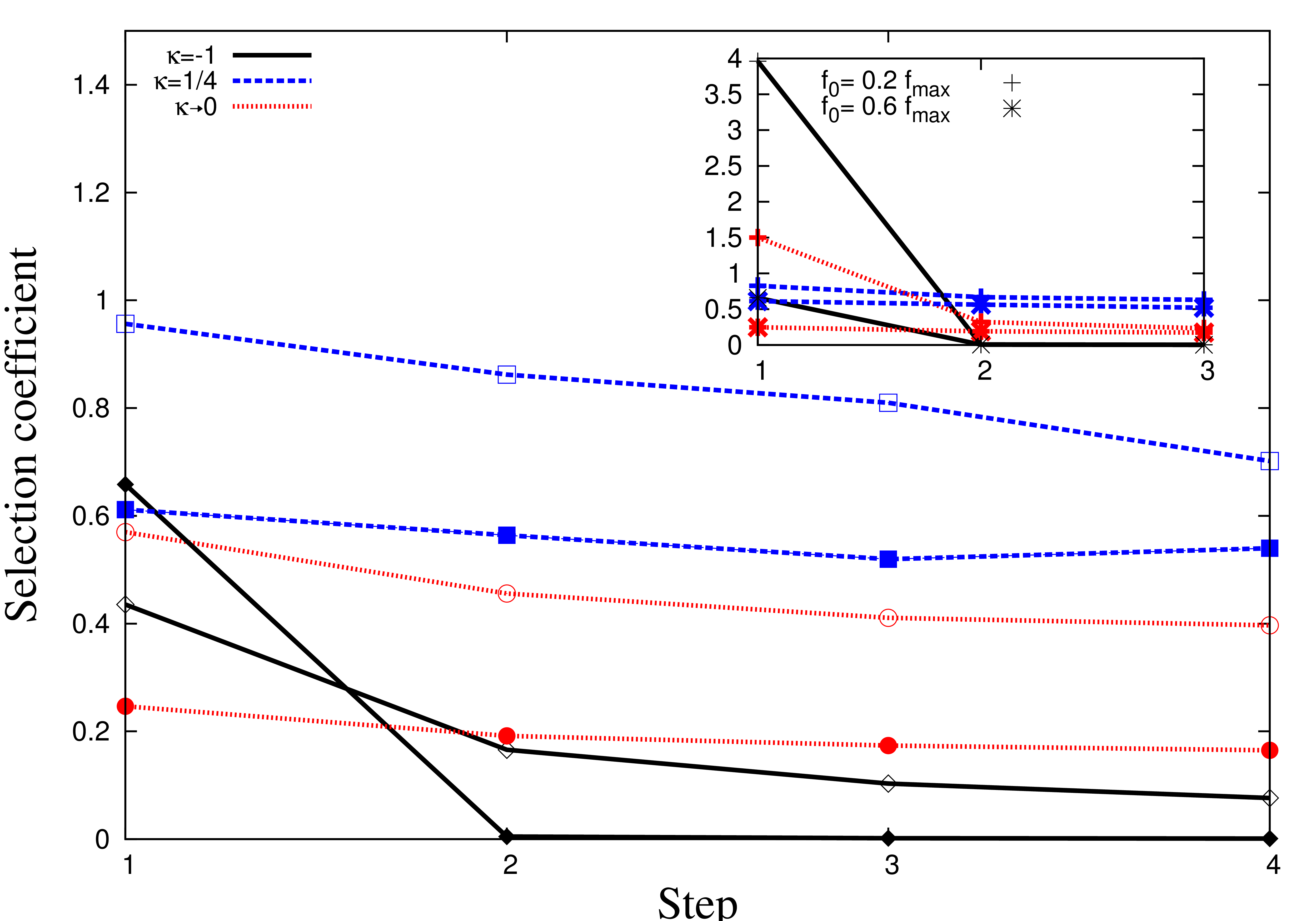

Supplement: S3 Fig — We considered two different Nμ where open symbols and closed symbols are for Nμ = 0.01 and Nμ = 50, respectively. The inset shows the selection coefficient of various steps for two different initial fitnesses f0 = 0.2fmax and f0 = 0.6fmax, where fmax is calculated using Eq (7) in the high mutation regime. (TIF) [file pone.0151795.s003.tif]
