# Supplementary material for: Correction: Exploiting the Adaptation Dynamics to Predict the Distribution of Beneficial Fitness Effects
Source: PLoS One. 2016 Nov 8;11(11):e0166503. doi: 10.1371/journal.pone.0166503 (PMC5100982; doi:10.1371/journal.pone.0166503)
Supplement: S1 File — (PDF) [file pone.0166503.s001.PDF]

RESEARCH ARTICLE

Exploiting the Adaptation Dynamics to Predict the Distribution of Beneficial Fitness Effects

Sona John*☯, Sarada Seetharaman☯

Theoretical Sciences Unit, Jawaharlal Nehru Centre for Advanced Scientific Research, Jakkur P.O., Bangalore 560064, India

☯ These authors contributed equally to this work.
* sonajohn@jncasr.ac.in

Abstract

Adaptation of asexual populations is driven by beneficial mutations and therefore the dynamics of this process, besides other factors, depends on the distribution of beneficial fitness effects. It is known that on uncorrelated fitness landscapes, this distribution can only be of three types: truncated, exponential and power law. We performed extensive stochastic simulations to study the adaptation dynamics on rugged fitness landscapes, and identified two quantities that can be used to distinguish the underlying distribution of beneficial fitness effects. The first quantity studied here is the fitness difference between successive mutations that spread in the population, which is found to decrease in the case of truncated distributions, remains nearly a constant for exponentially decaying distributions and increases when the fitness distribution decays as a power law. The second quantity of interest, namely, the rate of change of fitness with time also shows quantitatively different behaviour for different beneficial fitness distributions. The patterns displayed by the two aforementioned quantities are found to hold good for both low and high mutation rates. We discuss how these patterns can be exploited to determine the distribution of beneficial fitness effects in microbial experiments.

OPEN ACCESS

Citation: John S, Seetharaman S (2016) Exploiting the Adaptation Dynamics to Predict the Distribution of Beneficial Fitness Effects. PLoS ONE 11(3): e0151795. doi:10.1371/journal.pone.0151795

Editor: Frederick M. Cohan, Wesleyan University, UNITED STATES

Received: February 15, 2015

Accepted: March 4, 2016

Published: March 18, 2016

Copyright: © 2016 John, Seetharaman. This is an open access article distributed under the terms of the Creative Commons Attribution License, which permits unrestricted use, distribution, and reproduction in any medium, provided the original author and source are credited.

Data Availability Statement: All relevant data are within the paper and its Supporting Information files.

Funding: The authors have no support or funding to report.

Competing Interests: The authors have declared that no competing interests exist.

Introduction

Microbial populations have to constantly adapt in order to survive in a changing environment. For example, a bacterial population exposed to a new antibiotic must evolve in order to exist [1]. In asexual populations, this process of adaptation is driven only by rare beneficial mutations [2] which provide fitness advantage. Therefore, in order to survive in a new environment, enough beneficial mutations should be available and the beneficial mutations should confer sufficient fitness advantage. While the first factor depends on the mutation rate and population size, the second factor is determined by the underlying fitness distributions. Even though we have some understanding about the mutation rate of different microbial populations, blackthe full fitness distribution is more complex and relatively little is known about it. However, for

moderately adapted populations (i.e., fitness of the wild type is high enough), rare beneficial mutations which occur in the tail of the fitness distribution can be described by the extreme value theory (EVT) as proposed first by Gillespie [3]. The EVT states that the extreme tail of all distributions of uncorrelated random variables (fitness, in this case) can be of only three types. Depending on whether the tail of underlying fitness distribution is truncated or decaying faster than a power law or as a power law, the EVT distribution would belong to the Weibull or Gumbel or Fréchet domain, respectively [4]. All three EVT domains can be obtained from the generalized Pareto distribution given as

$$p(f) = (1 + \kappa f)^{-\frac{1+\kappa}{\kappa}}, \tag{1}$$

where κ is the tuning parameter. One example from each of the three EVT domains is shown in Fig 1, which shows the distribution of beneficial effects $p(f)$ with fitness f. The three types of EVT domains are classified according to the value of κ. Here negative κ belongs to the Weibull domain, while $\kappa = 0$ corresponds to the Gumbel domain and positive κ to the Fréchet domain. Interestingly, all three blackdistribution of beneficial fitness effects(DBFEs) have been observed in experiments on microbial populations [5–14]. While the exponential distribution belonging to the Gumbel domain has been most commonly seen [5–8], in recent times, the distribution of beneficial mutations belonging to the Weibull [10, 14] and Fréchet [11] domains have also been observed.

Recent theoretical studies have shown analytically and numerically that qualitatively different patterns occur in the adaptation dynamics of populations in different EVT domains of DBFEs in blacka low mutation regime [15–18]. Specifically, it has been shown that fitness gain in a fixation event follows the pattern of diminishing returns in the Weibull domain, constant returns in the Gumbel domain and accelerating returns in the Fréchet domain, and thus indicates that this quantity can be used to predict the DBFE. These observations are restricted to strong selection-weak mutation (SSWM) regime in which the genetic variation in the population is minimal, that is, only one beneficial mutation is present in the population in the time interval between its appearance and fixation [7]. It is then natural to ask whether the relationship between adaptation dynamics and the DBFE mentioned above are robust for large populations, where there might be more than one beneficial mutation competing for dominance in the population. The main aim of our study is to address this question and to see if the fitness gain in a fixation event can be used for predicting the DBFE in a more general scenario.

Here, we are mainly concerned with the populations in which a large number of mutants are produced at every generation. Hence, more than one beneficial mutation is expected to be present at the same time [19–23]. In this case, the beneficial mutations will compete with each other as has been observed in different experimental populations [24–27]. In this high mutation regime, as a result of the competition among the beneficial mutations, the rate of adaptation slows down. Fitness advantage due to the mutations that get fixed is much higher, since the availability of more mutations results in allowing only the best (fittest) mutation to get fixed [28]. A clear comparison of the population fraction of new mutants appearing in a population for two mutation regimes is given in Fig 2. In Fig 2(a) we see that the population in the SSWM regime is more or less monomorphic with only one mutant present at a time in all the three EVT domains. However, in a high mutation regime, the population is polymorphic with more than one mutant produced in it at every generation as shown in Fig 2(b). In fact, a large amount of genetic variation is observed in the case of bounded distributions corresponding to $\kappa < 0$ in Eq (1) resulting in a strong competition between the beneficial mutants.

In this work, we have used Wright-Fisher dynamics to study the adaptation dynamics of an asexual population in high and low mutation regimes for the three EVT domains of DBFE. The

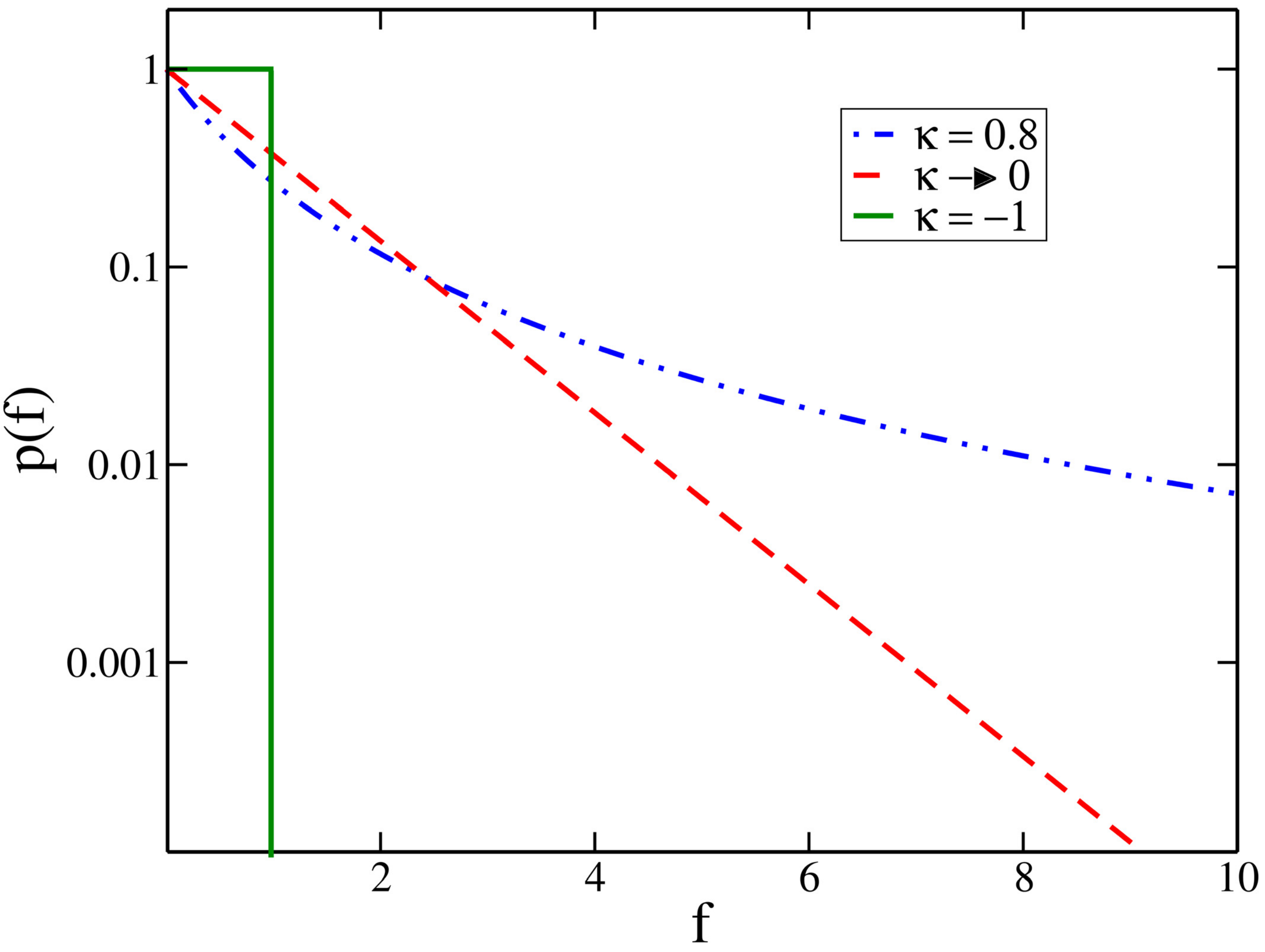

Fig 1. The figure shows the distribution of beneficial fitness effects *p*(*f*) with fitness *f* for the three EVT domains, given by Eq (1) for various *κ*. Here, κ is the tuning parameter with $\kappa > 0$, $\kappa \to 0$ and $\kappa < 0$ corresponding to the Fréchet, Gumbel and Weibull domains respectively.

doi:10.1371/journal.pone.0151795.g001

main motivation of this study is to look for quantities which can be used to distinguish between DBFEs using the properties of adaptation dynamics as opposed to the direct measurements of DBFEs. Our most important and interesting result is concerned with the fitness difference between mutations that spread in a population. This quantity shows qualitatively different trends in three EVT domains and thus helps in distinguishing the DBFEs.

We have also studied another quantity which is the rate of change of fitness with time, and observed that this shows quantitatively different behaviour for different EVT domains of the DBFEs. Though some results for the rate of change of fitness are already known in the literature [29], we measured it for all the three cases (Weibull, Gumbel and Fréchet) and identified that this can be used to distinguish the DBFEs in both SSWM and high mutation regimes. In order to obtain a complete picture, a comparison of our study with the existing literature is given in Table 1 below.

We also measured quantities like the genetic variation and the number of mutations in the most populated sequence. All of these quantities are discussed in the Results section. We suggest that the distinct trends shown by the above mentioned quantities can be used to predict

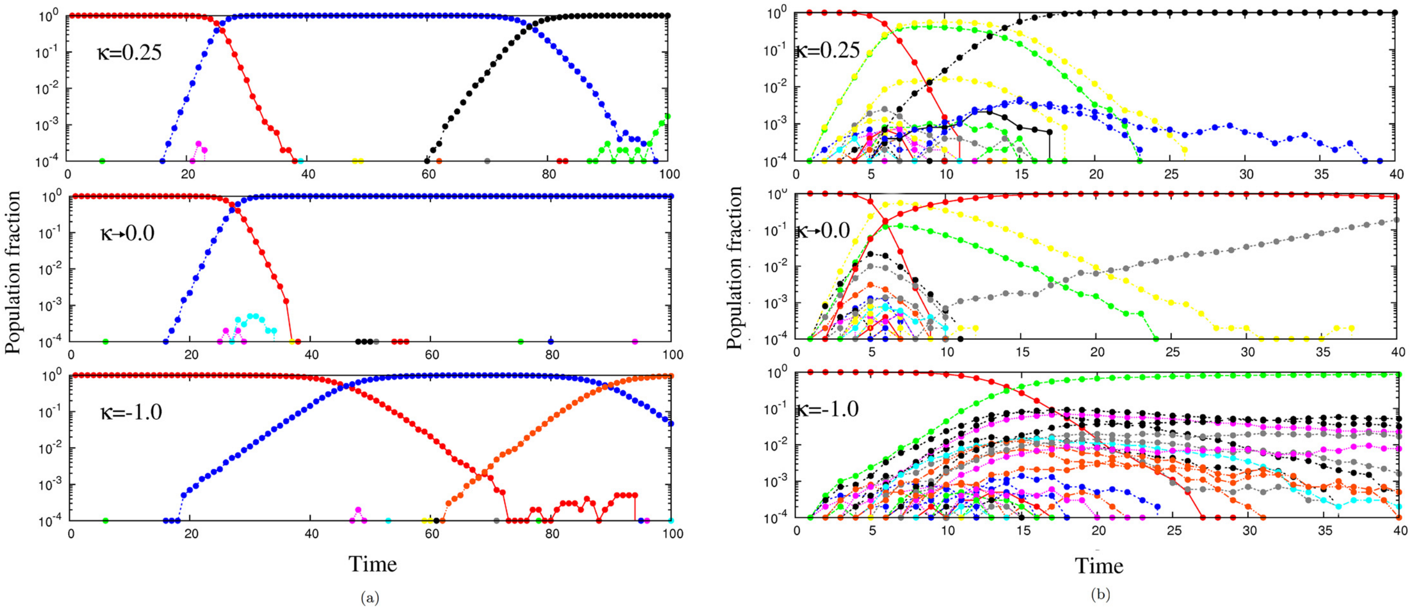

Fig 2. Population fraction of different mutant classes are shown as different coloured lines, where (a) shows the SSWM ($N\mu$ = 0.1, low mutation rate) regime and (b) shows the high mutation ($N\mu$ = 10) regime for all three EVT domains of DBFE.

doi:10.1371/journal.pone.0151795.g002

DBFEs from experimental studies on adaptation. The relevance of our work to experiments is also explored in the Discussion section.

Materials and Methods

We track the dynamics of a population of self-replicating (asexual), infinitely long binary sequences of fixed size using the standard Wright-Fisher process [21, 28]. In our work, the population size is held constant at $N = 10^4$, unless specified otherwise and the total mutation probability (beneficial and deleterious) per sequence is given by μ. Every occupied sequence is counted as a *class* and is labeled when it arises in the population. Initially, the whole population is in class 1 whose fitness is fixed and specified in every simulation run. We have used the term leader to refer to the class whose normalised probability of reproduction (product of population fraction and fitness) is greater than half. In that case, clearly class 1 is the initial leader since the whole population is localized there. At every time step, out of N sequences, m_t are chosen from a binomial distribution with mean $N\mu$ as mutants. Every mutant produced increases the number of classes in the population by one, and with time, the mutants may produce their own set of further mutants. The population fraction of each class may grow or go extinct, as can be observed in Fig 2. At any time t, the number of classes present in the population is given by $\mathcal{N}_c(t)$, and the population size and fitness of each class, i, where $1 \leq i \leq \mathcal{N}_c$, is

Table 1. Comparison with existing literature. Here, $\overline{\Delta f_{step}}$ is the average fitness difference between the present leader and the new beneficial mutation that gets established and $\bar{\mathcal{F}}(t)$ is the rate of change of fitness.

Quantities	DBFE domains: Low mutation regime			DBFE domains: High mutation regime		
	Weibull	Gumbel	Fréchet	Weibull	Gumbel	Fréchet
$\overline{\Delta f_{step}}$	[16]	[16]	[16]	this study	this study	this study
$\bar{\mathcal{F}}(t)$	this study	[29]	[29]	this study	[29]	this study

doi:10.1371/journal.pone.0151795.t001

denoted by $n(i, t)$ and $f(i)$, respectively. The normalized probability of each class at every time step, $\tilde{p}(i,t)$ contributing offspring to the population at the next time step, depends on the population size of the class at the present time step and the fitness of the class as

$$\tilde{p}(i,t) = \frac{n(i,t)\,f(i)}{\Sigma_{j=1}^{\mathcal{N}_c(t)} n(j,t) f(j)}. \tag{2}$$

Note that though the fitness of the class is the same as long as it persists in the population, its size may vary at every time step, thus changing its probability of reproduction as given by Eq (2). Different classes are populated in the next time step based on the multinomial distribution

$$P(n(1,t'), n(2,t')..n(\mathcal{N}_c,t')) = N! \prod_{j=1}^{\mathcal{N}_c(t)} \frac{[\tilde{p}(j,t)]^{n(j,t)}}{n(j,t)!}, \tag{3}$$

where $t' = t + 1$. The above equation is subject to the constraint $\Sigma_{j=1}^{\mathcal{N}_c(t)} n(j,t') = N$. In our simulations, we implement Eq (3) along with the above constraint by converting Eq (3) to a binomial distribution for every class, $1 \leq i < \mathcal{N}_c(t)$ as

$$n(i,t') = \binom{\tilde{N}(i)}{n(i,t)} q(i,t)^{n(i,t)} (1 - q(i,t))^{\tilde{N}(i) - n(i,t)}. \tag{4}$$

We set the population size of the last class as $n(\mathcal{N}_c(t), t') = N - \sum_{i=1}^{\mathcal{N}_c(t)-1} n(i,t')$. In Eq (4),

$$q(i,t) = \frac{\tilde{p}(i,t)}{\Sigma_{j=i}^{\mathcal{N}_c(t)} \tilde{p}(j,t)}, \tag{5}$$

and $\tilde{N}(i) = N - \Sigma_{j=1}^{i-1} n(j,t)$.

At every time step, once the classes are populated based on the algorithm described above, m_t sequences are chosen as mutants based on the binomial distribution with mean $N\mu$. Every new mutant class that appears in the population reduces the population size of the class in which it arose by one. In our work, we have varied μ to access both the SSWM (low mutation) and the high mutation regime. In our simulations unless specified otherwise, $N\mu = 0.01$ in low (SSWM) and $N\mu = 50$ in high mutation regimes.

A new class is assigned to each mutant and its fitness is chosen from a generalized Pareto distribution [4] given in Eq (1). The advantage of using Eq (1) is that we can access all three EVT domains of DBFE by changing κ. The distributions whose $\kappa < 0$ belong to the Weibull domain, while $\kappa = 0$ belong to the Gumbel domain, and $\kappa > 0$ belong to the Fréchet domain, respectively. The frequency distribution of beneficial effects $p(f)$ for various values of κ is shown in Fig 1. The upper bound u for the distributions chosen from Eq (1) is infinity when $\kappa \geq 0$ and equals $-1/\kappa$ for $\kappa < 0$. In this work, the fitness of the mutants is independently chosen from Eq (1) thus making the fitness of the mutant, F_m an uncorrelated variable, which may be greater or smaller than the parent fitness, F_p. We have analyzed the results to see how they vary between the three EVT domains and different mutation rates.

In the allocation of the fitness to any mutant, our work differs from the other works on clonal interference [21, 28] wherein the fitness of the mutant is hiked above the parent fitness by the selection coefficients (s) which may be held constant or chosen from a distribution as $F_m = (1 + s)F_p$. Unlike the model we have used in this work (as explained above), in this case, there is a strong correlation between the mutant fitness F_m and the parent fitness F_p. In those cases, the mutant fitness is always greater than the parent fitness and on an average, a double or higher mutant is fitter than a single mutant. This is in contrast with our work since in ours,

as the fitness of the parent increases, the number of better mutants available decreases thus producing different patterns for the fitness increment in each EVT domain.

In our model, whenever a mutant class goes extinct, the classes below it are moved up and the number of classes in the population is reduced by one. The normalised probability of reproduction given in Eq (2) of a class exceeding half corresponds to a leader change. The new leader determined now belongs to the class whose normalised probability exceeded half. We have also explored other criteria for defining the leader as the most populated class and find that our main results are robust with respect to the change in criteria (data not shown).

Every change of a leader is counted as a *step*. In the high mutation regime, the population is spread over many sequences and a sequence can produce two or more mutants, each of which may become leaders at different time steps. However, in the SSWM regime, the whole population is localised at a single sequence with a fixed fitness and can only move to a different sequence with higher fitness one mutation away. Thus every new leader arises from the previous leader, as can be observed in Fig 2(a). When a better sequence appearing in the population does not get lost due to genetic drift, it quickly gets fixed. Further mutations that may lead to future leaders appear in this genetic background. The change in the fitness of the population is the same as the change in the fitness of the leader. In this case, every move of the population (leader) from one sequence to another is termed as a step in the adaptive walk [30–33], whereas in the high mutation regime, the population is polymorphic and as seen from Fig 2(b) the leader change is not obvious.

Various quantities like the difference in fitness between successive leaders and the average number of mutations in the leader are averaged only over the walks that take the step. Other quantities like the number of classes present at any point in time and the rate of change of fitness are averaged over all time steps in that simulation run.

In this paper, the total number of iterations is 10^5 in every simulation run and the dynamics are tracked for a finite time limit of 10^4 generations, which we shall refer to as t_{max}. In this time span, the maximum fitness value, f_{max} that arises in the population can be calculated as

$$t_{max} N\mu \int_{f_{max}}^{u} p(f)df = 1, \quad (6)$$

where u is the upper limit of the fitness distribution equalling (-1/κ) for bounded distributions and infinity for the unbounded ones [4]. From the above integral, we get

$$f_{max} = \frac{(t_{max} N\mu)^{\kappa} - 1}{\kappa}. \quad (7)$$

Results

The number of classes in the population

For a population that is fixed in size, the number of classes in the population is expected to increase with the mutation rate. The average genetic variation, which is defined here as the average number of classes ($\mathcal{N}_c$) present in the population is shown in Fig 3 for all three DBFE domains. The top and bottom panels of the figure show the data corresponding to the high and low mutation regimes respectively. In both mutation regimes, we see that the average number of classes increase during the initial time steps and decrease at later times when the classes with lower fitness are eliminated by the fitter ones. The maximum number of classes existing in the population for the first case, as shown in Fig 3(a), does not belong to the lowest initial fitness, but to a slightly higher initial fitness. This could be because when the initial fitness is low, its

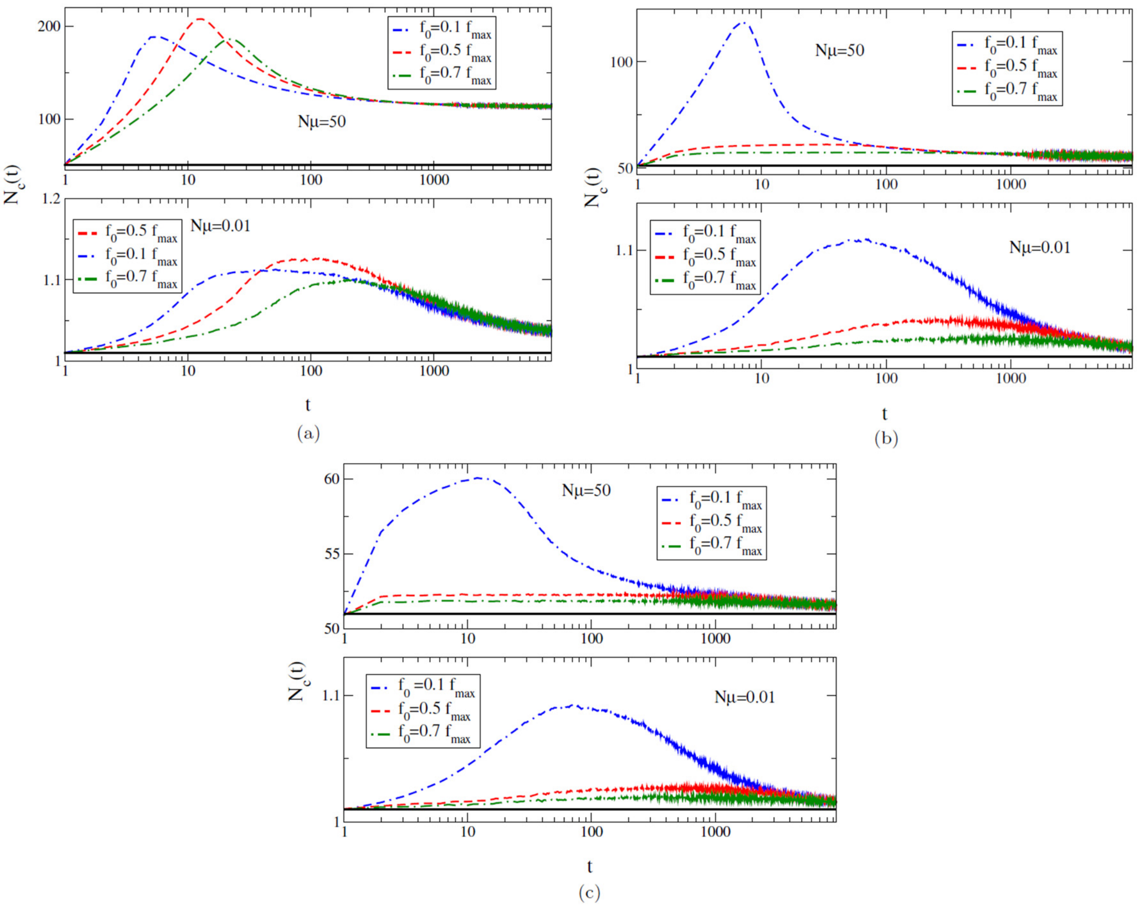

Fig 3. The plot shows the average number of classes in the population as function of time for various initial fitnesses. The fitnesses are chosen from Eq (1) with (a) $\kappa = -1$ (b) $\kappa \to 0$ and (c) $\kappa = 1/4$. For each κ value, the plot shows $\mathcal{N}_c(t)$ in both high mutation (top panels) and low mutation (bottom panels) regimes. The straight line in all plots shows $N\mu + 1$.

doi:10.1371/journal.pone.0151795.g003

class is quickly replaced by a fitter mutant and all further mutants that arise on this new background must compete with this fitter class.

In the low mutation regime, the population is localized at a single sequence for most of the time and produces $N\mu$ mutants at every time step. Hence, in this case, the average number of classes approach a constant $N\mu + 1$ at large times as can be seen in the bottom panels of Fig 3. These panels also indicate that the value of this constant increases with decreasing κ. This is because in the case of bounded distributions with $\kappa < 0$, the fitness of blacka beneficial mutant produced is expected to be closer to the parent fitness. In other words, mutations are nearly neutral and thus it takes a longer time to take over the population as shown in Fig 2(a). This results in blacka larger number of mutants in the Weibull domain, which can be observed in the bottom panel of Fig 3(a). We can clearly see from the top panels of Fig 3 that number of classes increases with decreasing κ even in a high mutation regime. Moreover, the average number of classes present at a time is much higher in this regime. This makes sense because the fitness of the classes belonging to $\kappa = -1$ cannot be very different from each other (can take on values between 0 and 1), which makes it possible for many of them to exist in the population. The maximum fitness of the classes belonging to $\kappa = 1/4$ distribution will on an average be much higher than all others (since the distribution is unbounded with a fat tail), thus outcompeting the others in the population.

Number of mutations in the leader

In the low mutation regime, the average number of mutations in the leader is expected to be very close to the step number since the genetic variation in the population is low and any mutation that escapes drift quickly takes over the population [3]. We verify this point via simulations as depicted in Fig 4. We find that the mutation number equals the step number in all the three EVT domains of the DBFE in the low mutation regime during the initial steps. However in the high mutation regime, the number of mutations in the leader of any step differs between the three DBFE domains. When the mutation rate is increased, the genetic variation of the population and the significance of clonal interference also increases. In the high mutation regime, the number of mutations in the leader is found to be less than the step number in all three DBFE domains. This is because there is a chance that different mutants originating from the same parent class can become the leader of the population at different times. This decrease from the step number is the minimum for the fat-tailed distributions and maximum for the truncated ones, as shown in Fig 4. This result is consistent with the number of classes present in the population as discussed in the previous section. In the Fréchet domain, since the clonal interference is minimal, it is most likely that a mutant originating from the present leader will become the next one. In the Weibull domain, due to the large number of classes present in the population, mutants originating from the same class can become leaders at different time points.

Fitness and fitness difference

From our simulations, we find that the average fitness of the first mutant fixed in the population, $\bar{f}_1$ increases linearly with initial fitness, f_0 for all κ in the low mutation regime and for $\kappa \neq 0$ in the high mutation regime. So we can write

$$\bar{f}_1 = a_\kappa^{(N\mu)} f_0 + b_\kappa^{(N\mu)}, \tag{8}$$

where the coefficients $a_\kappa^{(N\mu)}$ and $b_\kappa^{(N\mu)}$ are constants. In the low mutation regime, where the population for most times is monomorphic, the adaptive walk model has been used to analytically obtain the fitness at the first step, $\bar{f}_1$ as [15, 16]

$$\bar{f}_1 = \int_{f_0}^{u} df \;\, T(f \leftarrow f_0) f, \tag{9}$$

where the transition probability

$$T(f \leftarrow f_0) = \frac{\left(1 - e^{-\frac{2(f-f_0)}{h}}\right) p(f)}{\int_{f_0}^{u} dg \left(1 - e^{-\frac{2(g-f_0)}{f_0}}\right) p(g)}. \tag{10}$$

In this model, from Eq (9), the coefficient $a_\kappa^{(N\mu \ll 1)}$ was obtained as 0.33, 1.0 and 1.6 for $\kappa = -1$, 0, and 1/4, respectively. The corresponding $b_\kappa^{(N\mu \ll 1)}$ for the aforementioned κ were 0.66, 2.0 and 1.89 [16]. In the high mutation regime where the adaptive walk model is not applicable, we obtained the values for the coefficients in Eq (8) numerically. We find that for large f_0, $a_\kappa^{(50)}$ equals 0.004 and 1.5 and $b_\kappa^{(50)}$ equals 0.99 and 9.1 for $\kappa = -1$ and 1/4 respectively.

The interesting result from our work is that, irrespective of the number of mutants produced in the population, the difference $\overline{\Delta f_{step}} = \bar{f}_1 - f_0$ between the fitness of the first step and the initial fitness displays different qualitative trends: it increases for positive κ, approaches a constant when $\kappa = 0$ and decreases for negative κ as shown in Fig 5 and S1 Fig.

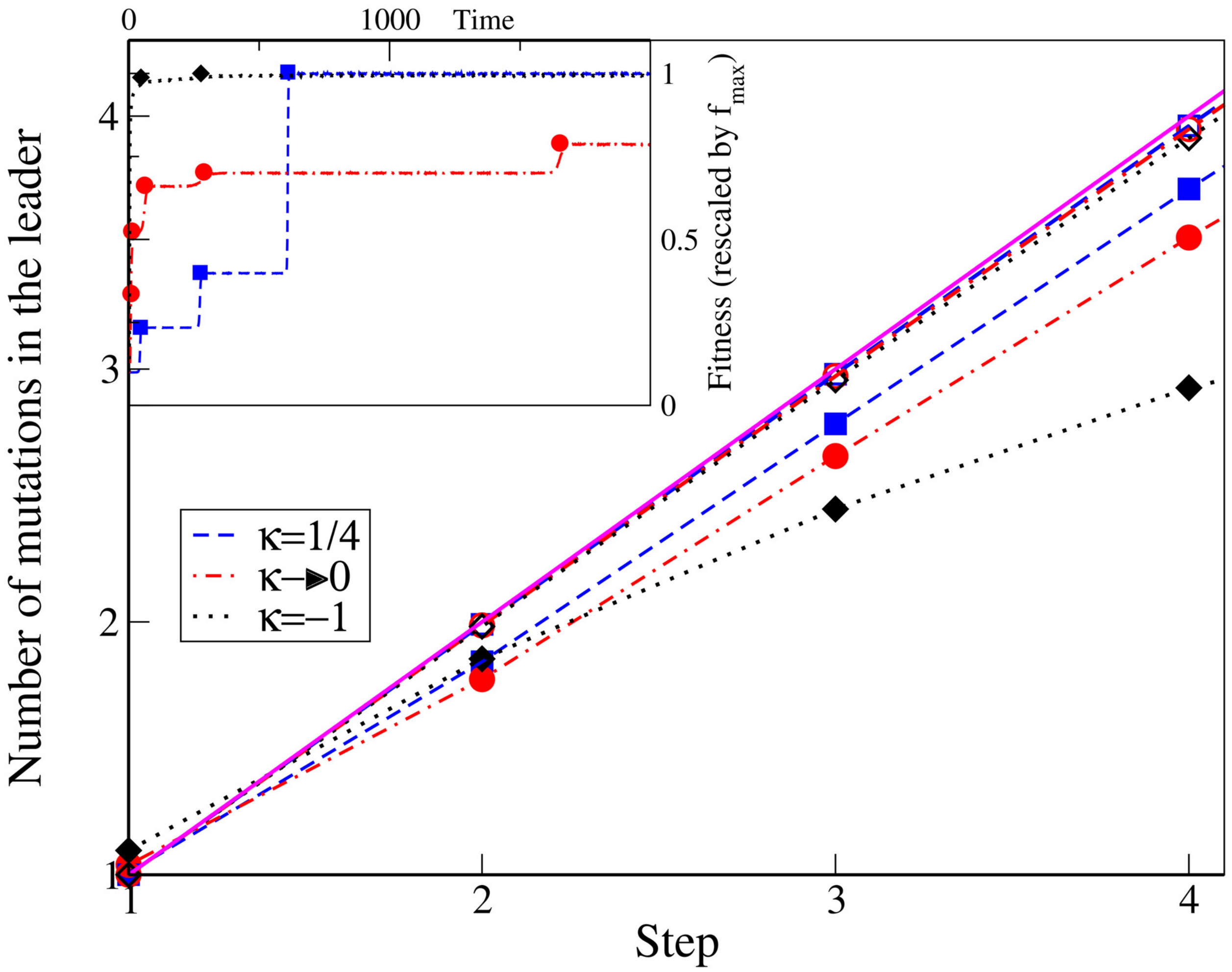

Fig 4. The main plot shows the number of mutations in the leader at any step for various *κ* and mutation rates. The simulation data is represented by points while the broken lines connect the data points. The solid line shows $y = x$. In the inset, from a single simulation run, the fitness of the whole population as a function of time is shown by broken lines and the fitness of the leader, whenever the leader changes, is shown in symbols.

doi:10.1371/journal.pone.0151795.g004

We can better understand these increasing and decreasing trends by the following heuristic argument. In both the low and high mutation regimes, for large f_0, the fitness at the first step f_1 increases linearly with the initial fitness is given in Eq (8). Therefore, we can write the selection coefficient defined as the relative fitness difference at the first step as

$$s = \frac{\bar{f}_1 - f_0}{f_0} = \frac{(a_\kappa^{(N\mu)} - 1)f_0}{f_0} + \frac{b_\kappa^{(N\mu)}}{f_0}, \quad \text{for all} \quad \kappa\ , N\mu. \tag{11}$$

In an adapting population, since the fitness of the first step is greater than the initial fitness, the selection coefficient is always positive. As the fitness distributions belonging to the Fréchet domain are unbounded with fat tails, high f_0 values can be considered. In this case, the second term on the right hand side (RHS) of Eq (11) can be ignored and we can write $s \approx (a_\kappa^{(N\mu)} - 1) > 0$. Thus for $\kappa > 0$, since $a_\kappa^{(N\mu)} > 1$ it follows that the fitness difference at the first step increases with f_0. On the other hand, since the distribution belonging to the Weibull domain is truncated, we can invoke the following inequality to explain the decrease in fitness

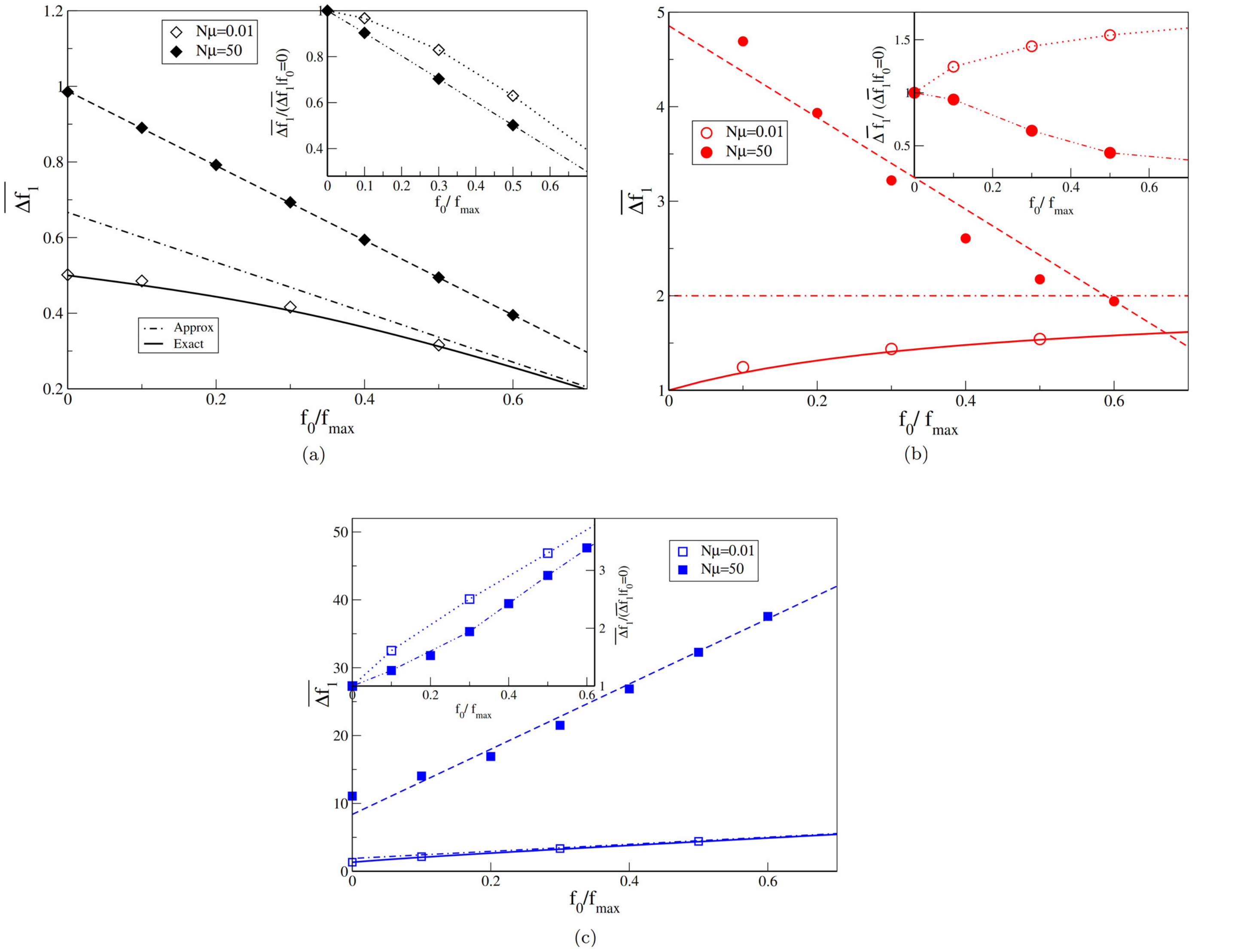

Fig 5. The main plot shows the fitness difference at the first step as a function of the initial fitness for various *Nμ*. The fitnesses are chosen from Eq (1) with (a) $\kappa = -1$ (b) $\kappa \to 0$ and (c) $\kappa = 1/4$. The solid lines in the main plot are obtained by numerically evaluating the integral given by Eq (9), while the dotted lines are the approximate results that can be obtained for the results when the initial fitness is high in the low mutation regime. The broken lines for $\kappa \neq 0$ are lines of best fit as mentioned in the text. The broken line for $\kappa \to 0$ is used for connecting the data points. The inset shows the fitness difference at the first step as a comparative measure of the fitness difference obtained at the first step when $f_0 = 0$. Here, the lines are used for connecting the data points.

doi:10.1371/journal.pone.0151795.g005

difference with increasing f_0:

$$\bar{f}_1 - f_0 < u - f_0, \tag{12}$$

where u is the upper limit of the fitness distribution. With increasing f_0, the RHS of the above equation decreases showing that as the initial fitness increases, $\bar{f}_1 - f_0$ has to necessarily decrease. Thus, the qualitative trends discussed above appear to be determined by the behaviour of the tail (bounded/unbounded), and not by the details of the model.

Further, it is interesting to note that while the data points for the exponentially decaying distribution ($\kappa = 0$) increase and seem to be approaching a constant in the low mutation regime, the data in the high mutation regime seems to be reducing to approach the same constant. Our

simulation results shown in Fig 5 not only match the predicted theoretical values and validate the claim of different qualitative trends in each EVT domain in the SSWM regime, but also show that the trends hold irrespective of the number of mutants produced in the population. This result suggests that the qualitatively different trends of the fitness difference (increasing, constant and decreasing with initial fitness in the Fréchet, Gumbel and Weibull domains, respectively), can be used to distinguish between the EVT domains in a more general scenario.

Though the fitness difference at the first step is greater in the high mutation regime, when compared with the results in the low mutation regime, when we look at the fitness difference at the first step scaled by the fitness difference obtained when the initial fitness is zero (insets of Fig 5), we see that this increase is slower in the high mutation regime compared to the results obtained in the low mutation regime. This indicates that as the mutation rate increases, though the number of mutants accessed is higher, the difference in fitness compared to a lower initial fitness is not proportionally higher and is in fact lower for all the fitness distributions.

Rate of change of fitness with time

Besides the fitness increment at a fixed event of leader change, we also measured the fitness as a function of time as shown in Fig 6. We observed that even though the fitness increases with time in all the three EVT domains, the rate at which the fitness increases depends strongly on the DBFE. This rate has an initial fast transient phase, after which it slows down.

The initial transient phase is strongly dependent on the initial condition as well as the mutation rate as shown in S2 Fig. The increase in fitness is fastest for the lowest initial condition, but it approaches the same fitness value as in the case of higher initial fitness in few generations. The time taken for populations of different initial fitness to reach the same fitness value depends on the mutation rate: for $N\mu \gg 1$, it takes about 20 generations, whereas for $N\mu \ll 1$,

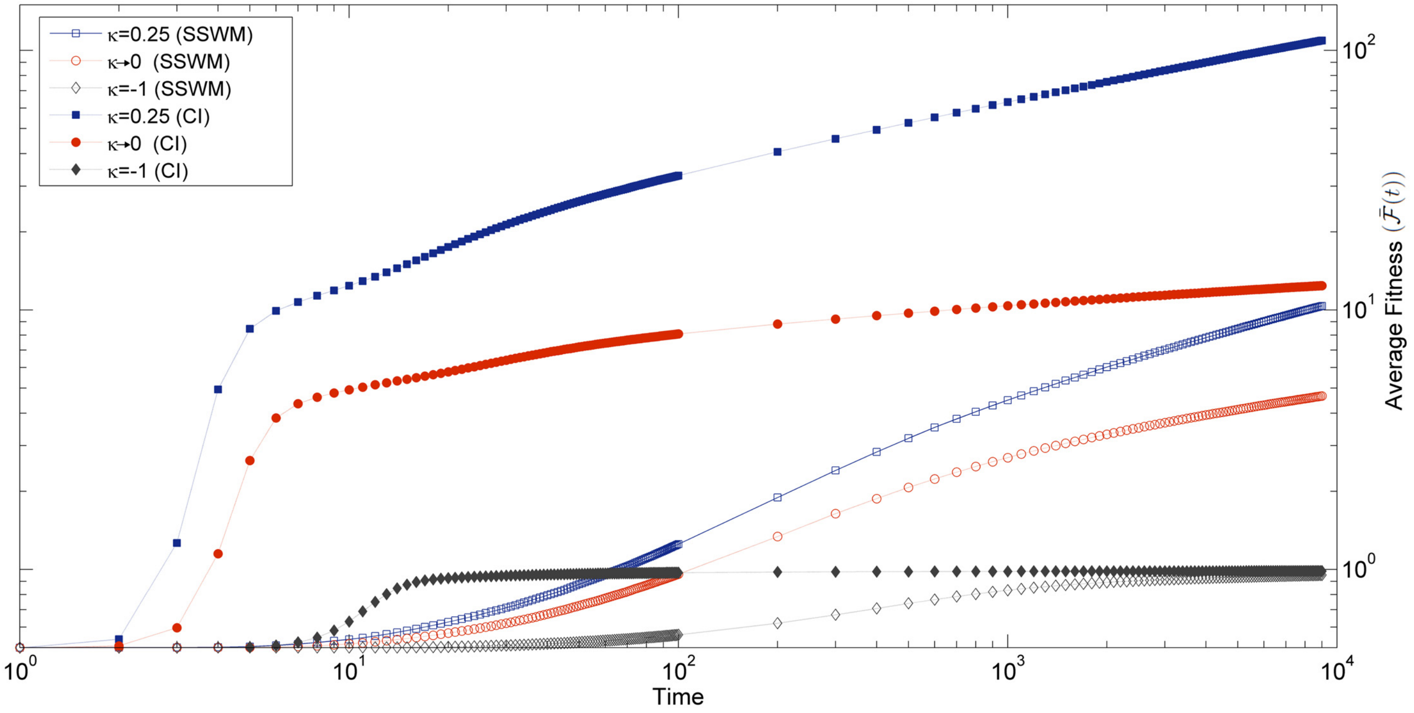

Fig 6. Figure shows the average fitness increase with time for three different values of κ in the SSWM regime ($N\mu = 0.01$), and in the high mutation regime($N\mu = 50$). In all the cases, population starts with the same initial fitness $f_0 = 0.5$.

doi:10.1371/journal.pone.0151795.g006

it is approximately 200 generations. Even after this transient phase, the rate of increase in average fitness ($\bar{\mathcal{F}}(t)$) with time depends on the mutation rate as shown in Fig 6. This is because of the fact that when blacka large number of mutations is available at the same time, a highly fit mutant can invade the population and give a large fitness increment. Therefore, the fitness of a highly fit mutant sequence would be greater in the high mutation regime compared to the one in a low mutation regime. The maximum fitness value reached in 9000 generations, in the case of Fréchet distribution, is about 10 times more for the high mutation regime, which is consistent with the expectation from Eq (7). Even beyond this point we noticed that the fitness is still increasing. In the same way, the Gumbel distribution also shows a significant increase in maximum fitness reached in the high mutation regime as compared to the SSWM regime (about 4 times). Here also we found that the fitness is still increasing beyond the time point till which we tracked the dynamics. The bounded distribution (Weibull) reaches near the upper bound in SSWM and evolves slowly. However, fitness reaches a fitness plateau in the high mutation regime and rate of adaptation becomes zero as can be seen in Fig 6.

From this, we observe that the rate of change of fitness strongly depends on the properties of the underlying DBFE, which suggests that looking at this quantity can help us in distinguishing the DBFEs. Hence, we measured the fitness increment defined as

$$\Delta\bar{\mathcal{F}}(t) = \langle\bar{\mathcal{F}}(t+1) - \bar{\mathcal{F}}(t)\rangle, \tag{13}$$

at each step. The $\Delta\bar{\mathcal{F}}(t)$ initially increases, then slowly decreases and settles down to a zero as shown in Fig 7. If we denote this function as

$$\Delta\bar{\mathcal{F}}(t) = \frac{A}{t^{\alpha}}, \tag{14}$$

where A is a constant and the exponent α can be used to distinguish the DBFE, since, as explained below, exponent α is found to be greater (smaller) than one in the Weibull (Fréchet) domain, but is close to one in the Gumbel domain.

In the SSWM regime, from Fig 7(a), we can see that each type of DBFE considered shows a different rate of decay. The Weibull domain has a faster decay with $\alpha = 1.86$, the Gumbel domain has $\alpha \approx 1$ [29] and the Fréchet domain $\alpha = 0.66$ [29]. We observed that the same trend is robust in blacka high mutation rate regime as well, where α values are slightly larger in all cases. In this regime also $\alpha = 2.02$, 1 and 0.76 for the Weibull, Gumbel and Fréchet domains, respectively as shown in Fig 7(b). In the high mutation regime, in the case of Weibull distributions, fitness reaches a plateau in few generations, after which its rate of change goes to zero as observed in Fig 7(b). The theoretical prediction for fitness at every time step for the unbounded distributions belonging to the Gumbel and Frèchet domains was obtained by Park and Krug [29] in the low mutation regime. The comparison of our simulation data with these predictions shows a very good agreement in the Gumbel domain and in the Fréchet domain (up to a constant). In this work, we have also considered the bounded distribution and observed that its rate of decrease is faster with an exponent greater than one, which was not considered in the previous studies. We observed that even in blacka high mutation regime, the exponent α shows the same behaviour. In this regime, the rate of change of fitness has been calculated only for exponential distribution belonging to the Gumbel domain [29] and their prediction matches with our data. In this work, we have obtained a complete picture by studying the rate of change of fitness numerically for the other two EVT domains as well.

Thus, the second main finding from our study is that in all DBFEs, the fitness difference at each time step decreases with time as given by Eq (14) and we can distinguish between the three EVT domains of DBFEs by looking at the exponent α.

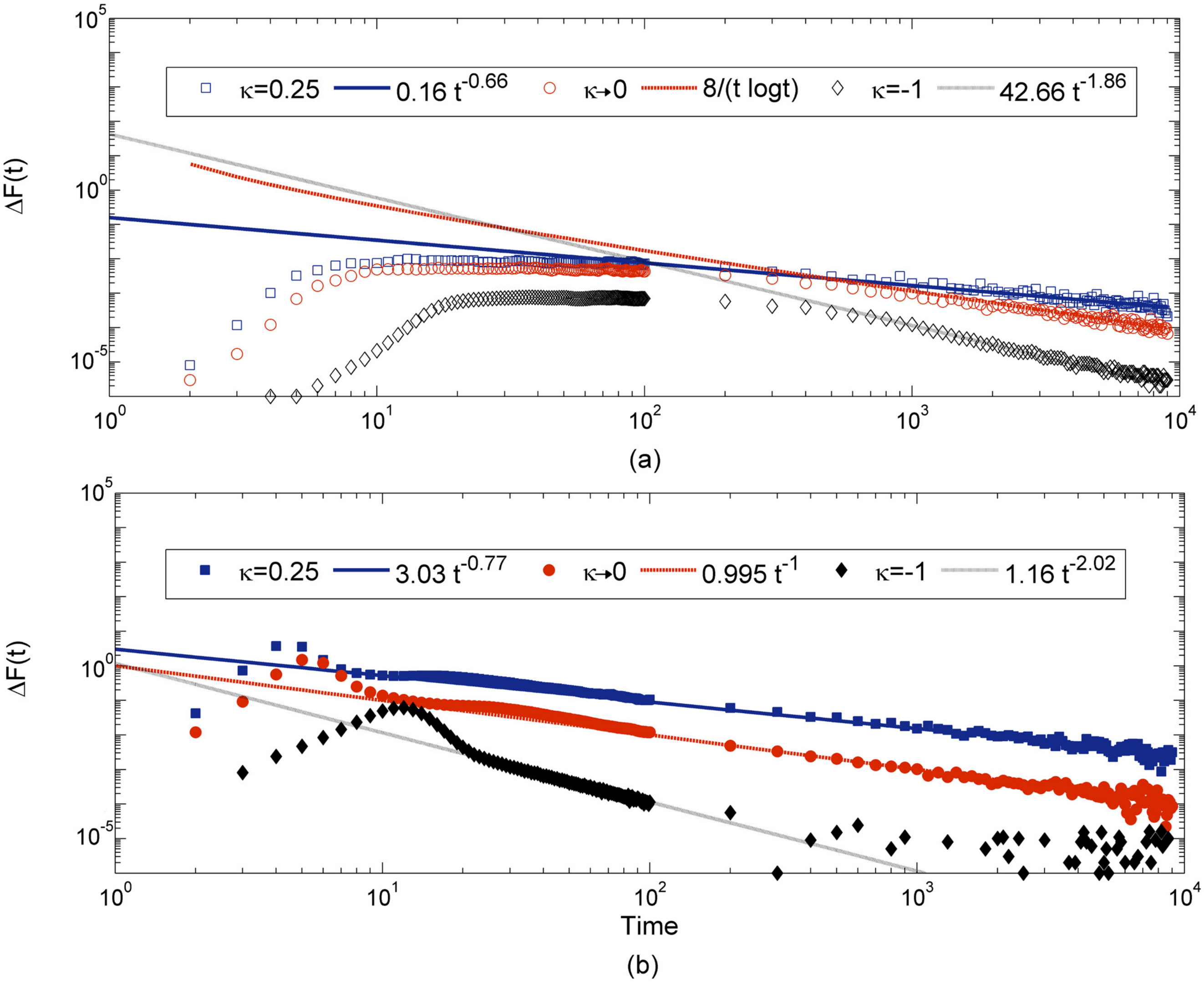

Fig 7. Figure shows the fitness increment in each time step for three different values of *κ* in two mutation regimes (SSWM and high mutation). In each case the data is fitted with the theoretically expected function given in Eq (14), except for blackthe exponential distribution for which we used the theoretical prediction by Park and Krug [29]. In all cases, the population starts with the same initial fitness $f_0 = 0.5$.

doi:10.1371/journal.pone.0151795.g007

Discussion

The main purpose of our work is to determine the quantities that can be used to distinguish the different extreme value domains of DBFE. Previous studies [16, 18] have found that in an adapting population, the fitness gain at each fixation event shows qualitatively different trends in the three DBFE domains when the number of mutants produced in the population is much less than one at every generation ($N\mu \ll 1$). The focus of this work is to explore the parameter regime in which the number of mutants produced is much above one ($N\mu \gg 1$). When the mutation rate is high, the population becomes polymorphic and the better mutants existing in the population compete with each other. From our study, we have observed that the qualitative trends found for fitness difference when a new mutation establishes in the low mutation regime hold irrespective of the number of mutants produced. Thus, this study suggests that blackthe

fitness difference between successive mutations that spread in the population is a very important and robust quantity that can be used to predict the DBFEs in a more general scenario.

From our simulations, we see that as the initial fitness is increased black, the fitness difference at the first step given by $\overline{\Delta f_{step}}$ reduces, approaches a constant black, or increases with blackthe initial fitness in the Weibull, Gumbel and Fréchet domains, respectively. We can understand these trends by a heuristic reasoning as discussed in detail in the Results section. This argument explains the increase in $\overline{\Delta f_{step}}$ with f_0 for blackan unbounded power law distribution and shows that the trends are determined by the behaviour of the tail (bounded/unbounded), and not by the details of the model.

Another important measure in understanding the dynamics of adaptation is the rate at which it occurs. Most of the previous studies which measured the adaptation rate have only considered exponentially distributed fitness distributions [20–22, 28, 34]. A previous study by Park and Krug [29] also considered DBFEs belonging to the Fréchet domain, but only in the SSWM regime (see Table 1). In this work, we have extended the previous studies by numerically measuring the rate of change of fitness for bounded distributions as well. We have measured the rate of change of fitness in all the three EVT domains of the DBFE in both low and high mutation regimes. We observed that in all the cases, the rate of change of fitness decreases with time as $\sim t^{-\alpha}$, where $\alpha > 1$ for Weibull, $\alpha \approx 1$ for Gumbel [29] and $\alpha < 1$ for Fréchet domains [29].

Experimentally, the distribution of beneficial fitness effects can be inferred by two methods. In the first method, mutations are introduced in the wild type sequence and those that confer a fitness advantage are separated and their distribution of fitness effects are determined. In this method, DBFE belonging to all the EVT domains have been observed [5–14]. In contrast, here we focus on learning about DBFE via adaptation dynamics. Though many works have tracked the dynamics of the population during adaptation [7, 35–38], in most of them only the selection coefficient of the mutant fixed was measured. In our study, we have observed that the selection coefficient as given by Eq (11) always decreases, with the increasing initial fitness or increasing steps as shown in S3 Fig. Hence, this quantity is not useful to distinguish between the EVT domains. However, from our study we observe that the fitness difference between steps shows different patterns depending on the EVT domain of the DBFEs in both the high and low mutation regimes and can be used to distinguish between the EVT domains.

In this work, we have numerically shown that the fitness returns in each EVT domain is very robust and holds good even when the number of mutations produced is large ($N\mu \gg 1$). Fitness difference can be measured in experiments, for example as in [5]. We suggest that experiments can predict the EVT domain of DBFE by measuring the fitness difference between successive mutations fixed in the population or even from the fitness of the first mutation, when the initial fitness is varied. However, currently experimental studies that measure both fitness and DBFE in the same study are not available, but it is highly desirable to have such studies to test our predictions.

Supporting Information

S1 Fig. The plot shows the fitness difference at the first step as a function of the initial fitness for different κ and two different $N\mu$. The lines give the theoretical values while the open symbols are the simulation output for $N\mu = 0.02$ and the closed symbols are those for $N\mu = 5$. (TIF)

S2 Fig. The figure shows the average fitness of the population for various κ in both the low and high mutation regimes. Two different initial conditions $f_0 = 0$ (open symbols) and $f_0 = 0.5$

(closed symbols) are considered.
(TIF)

S3 Fig. The main figure shows the selection coefficient as a function of step for all three κ values. We considered two different $N\mu$ where open symbols and closed symbols are for $N\mu = 0.01$ and $N\mu = 50$, respectively. The inset shows the selection coefficient of various steps for two different initial fitnesses $f_0 = 0.2f_{max}$ and $f_0 = 0.6f_{max}$, where f_{max} is calculated using Eq (7) in the high mutation regime.
(TIF)

Acknowledgments

We thank K. Jain for many useful discussions that helped us in this work and suggesting the heuristic argument discussed in the section 'Fitness and fitness difference'. We also thank J. Krug for bringing references [12, 13] to our attention. We thank V. Yalasi for helping us to improve the figure quality.

Author Contributions

Conceived and designed the experiments: SJ SS. Performed the experiments: SJ SS. Analyzed the data: SJ SS. Contributed reagents/materials/analysis tools: SJ SS. Wrote the paper: SS SJ.

References

1. Bull JJ, Otto SP (2005) The first steps in adaptive evolution. Nat Genet 37: 342–343. doi: 10.1038/ng0405-342 PMID: 15800646
2. Eyre-Walker A, Keightley P (2007) The distribution of fitness effects of new mutations. Nat Rev Genet 8: 610. doi: 10.1038/nrg2146 PMID: 17637733
3. Gillespie JH (1983) A simple stochastic gene substitution process. Theor Popul Biol 23: 202–215. doi: 10.1016/0040-5809(83)90014-X PMID: 6612632
4. Sornette D (2000) Critical Phenomena in Natural Sciences. Springer, Berlin.
5. MacLean RC, Buckling A (2009) The distribution of fitness effects of beneficial mutations in *Pseudomonas aeruginosa*. PLoS Genetics 5: e1000406. doi: 10.1371/journal.pgen.1000406 PMID: 19266075
6. Sanjuán R, Moya A, Elena S (2004) The distribution of fitness effects caused by single-nucleotide substitutions in an RNA virus. Proc Natl Acad Sci USA 101: 8396–8401. doi: 10.1073/pnas.0400146101 PMID: 15159545
7. Rokyta D, Joyce P, Caudle S, Wichman H (2005) An empirical test of the mutational landscape model of adaptation using a single-stranded DNA virus. Nat Genet 37: 441–444. doi: 10.1038/ng1535 PMID: 15778707
8. Kassen R, Bataillon T (2006) Distribution of fitness effects among beneficial mutations before selection in experimental populations of bacteria. Nat Genet 38: 484–488. doi: 10.1038/ng1751 PMID: 16550173
9. Rokyta DR, Beisel CJ, Joyce P, Ferris MT, Burch CL, et al. (2008) Beneficial fitness effects are not exponential for two viruses. J Mol Evol 69: 229.
10. Bataillon T, Zhang T, Kassen R (2011) Cost of adaptation and fitness effects of beneficial mutations in *Pseudomonas fluorescens*. Genetics 189: 939–949. doi: 10.1534/genetics.111.130468 PMID: 21868607
11. Schenk MF, Szendro IG, Krug J, de Visser JAGM (2012) Quantifying the adaptive potential of an antibiotic resistance enzyme. PLoS Genet 8: e1002783. doi: 10.1371/journal.pgen.1002783 PMID: 22761587
12. Foll M, Poh YP, Renzette N, Ferrer-Admetlla A, Bank C, et al. (2014) Influenza virus drug resistance: A time-sampled population genetics perspective. PLoS Genet 10(2). doi: 10.1371/journal.pgen.1004185 PMID: 24586206
13. Bank C, Ryan TH, Jeffrey DJ, Daniel N (2014) A systematic survey of an intragenic epistatic landscape. Mol Biol Evol. doi: 10.1093/molbev/msu301 PMID: 25371431

14. Rokyta DR, Abdo Z, Wichman HA (2009) The genetics of adaptation for eight microvirid bacteriophages. J Mol Evol 69: 229. doi: 10.1007/s00239-009-9267-9 PMID: 19693424
15. Jain K, Seetharaman S (2011) Multiple adaptive substitutions during evolution in novel environments. Genetics 189: 1029–1043. doi: 10.1534/genetics.111.134163 PMID: 21900275
16. Seetharaman S, Jain K (2014) Adaptive walks and distribution of beneficial fitness effects. Evolution 68: 965–975. doi: 10.1111/evo.12327 PMID: 24274696
17. Seetharaman S, Jain K (2014) Length of adaptive walk on uncorrelated and correlated fitness landscapes. Phys Rev E 90: 32703. doi: 10.1103/PhysRevE.90.032703
18. Seetharaman S (2011) Adaptation on rugged fitness landscapes. M. S. thesis, JNCASR, Bangalore.
19. Muller HJ (1964) The relation of recombination to mutational advance. Mutation Res 1: 2–9. doi: 10.1016/0027-5107(64)90047-8
20. Gerrish PJ, Lenski RE (1998) The fate of competing beneficial mutations in an asexual populations. Genetica 102: 127–144. doi: 10.1023/A:1017067816551 PMID: 9720276
21. Park SC, Krug J (2007) Clonal interference in large populations. PNAS 104: 18135–18140. doi: 10.1073/pnas.0705778104 PMID: 17984061
22. Desai M, Fisher D (2007) Beneficial mutation-selection balance and the effect of linkage on positive selection. Genetics 176: 1759–1798. doi: 10.1534/genetics.106.067678 PMID: 17483432
23. Jain K, Krug J, Park SC (2011) Evolutionary advantage of small populations on complex fitness landscapes. Evolution 65–7: 1945–1955. doi: 10.1111/j.1558-5646.2011.01280.x
24. de Visser JAGM, Rozen DE (2006) Clonal interference and the periodic selection of new beneficial mutations in escherichia coli. Genetics 172: 2093–2100. doi: 10.1534/genetics.105.052373 PMID: 16489229
25. de Visser JAGM, Zeyl C, Gerrish P, Blanchard J, Lenski R (1999) Diminishing returns from mutation supply rate in asexual populations. Science 283: 404–406. doi: 10.1126/science.283.5400.404
26. Miralles R, Gerrish PJ, Moya A, Elena S (1999) Clonal interference and the evolution of rna viruses. Science 285: 813–815. doi: 10.1126/science.285.5434.1745
27. Rozen D, de Visser JAGM, Gerrish PJ (2002) Fitness effects of fixed beneficial mutations in microbial populations. Curr Biol 12: 1040–1045. doi: 10.1016/S0960-9822(02)00896-5 PMID: 12123580
28. Park SC, Simon D, Krug J (2010) The speed of evolution in large asexual populations. J Stat Phys 138: 381–410. doi: 10.1007/s10955-009-9915-x
29. Park SC, Krug J (2008) Evolution in random fitness landscapes: the infinite sites model. J Stat Mech: Theor Exp 2008: P04014. doi: 10.1088/1742-5468/2008/04/P04014
30. Wilke C, Martinetz T (1999) Adaptive walks on time-dependent fitness landscapes. Phys Rev E 60: 2154–2159. doi: 10.1103/PhysRevE.60.2154
31. Orr HA (2003) The distribution of fitness effects among beneficial mutations. Genetics 163: 1519–1526. PMID: 12702694
32. Rosenberg N (2005) A sharp minimum on the mean number of steps taken in adaptive walks. J theor Biol 237: 17–22. doi: 10.1016/j.jtbi.2005.03.026 PMID: 15979094
33. Kryazhimskiy S, Tkačik G, Plotkin JB (2009) The dynamics of adaptation on correlated fitness landscapes. Proc Natl Acad Sci USA 106: 18638–18643. doi: 10.1073/pnas.0905497106 PMID: 19858497
34. Campos P, Wahl LM (2010) The adaptation rate of asexuals: deleterious mutations, clonal interference and population bottlemecks. Evolution 64(7): 1973–1983. PMID: 20199567
35. Schoustra S, Bataillon T, Gifford D, Kassen R (2009) The properties of adaptive walks in evolving populations of fungus. PLoS Biol 7 (11): e1000250. doi: 10.1371/journal.pbio.1000250 PMID: 19956798
36. MacLean RC, Perron GG, Gardner A (2010) Diminishing returns from beneficial mutations and pervasive epistasis shape the fitness landscape for rifampicin resistance in *Pseudomonas aeruginosa*. Genetics 186: 1345–1354. doi: 10.1534/genetics.110.123083 PMID: 20876562
37. Gifford DR, Schoustra SE, Kassen R (2011) The length of adaptive walks is insensitive to starting fitness in *Aspergillus nidulans*. Evolution 65: 3070–3078. doi: 10.1111/j.1558-5646.2011.01380.x PMID: 22023575
38. Sousa A, Magalhães S, Gordo I (2012) Cost of antibiotic resistance and the geometry of adaptation. Mol Biol Evol 29: 1417–1428. doi: 10.1093/molbev/msr302 PMID: 22144641
